# WASH Benefits Bangladesh trial: system for monitoring coverage and quality in an efficacy trial

**DOI:** 10.1186/s13063-018-2708-2

**Published:** 2018-07-06

**Authors:** Mahbubur Rahman, Sania Ashraf, Leanne Unicomb, A. K. M. Mainuddin, Sarker Masud Parvez, Farzana Begum, Kishor Kumar Das, Abu Mohd. Naser, Faruqe Hussain, Thomas Clasen, Stephen P. Luby, Elli Leontsini, Peter J. Winch

**Affiliations:** 1 Environmental Intervention Unit, Enteric and Respiratory Disease Program, Infectious Disease Division, International Centre for Diarrheal Disease Research, Bangladesh (icddr,b), Dhaka, Bangladesh; 2 Johns Hopkins Bloomberg School of Public Health, Baltimore, MD USA; 3 Rollins School of Public Health, Emory University, Atlanta, GA USA; 4 Division of Infectious Diseases and Geographic Medicine, Stanford University, Stanford, CA USA

**Keywords:** WASH Benefits, Implementation fidelity, Water quality, Sanitation, Handwashing, Child nutrition, Efficacy, Behavior change, Cluster randomized controlled trial, Bangladesh

## Abstract

**Background:**

Researchers typically report more on the impact of public health interventions and less on the degree to which interventions were followed implementation fidelity. We developed and measured fidelity indicators for the WASH Benefits Bangladesh study, a large-scale efficacy trial, in order to identify gaps between intended and actual implementation.

**Methods:**

Community health workers (CHWs) delivered individual and combined water, sanitation, handwashing (WSH) and child nutrition interventions to 4169 enrolled households in geographically matched clusters. Households received free enabling technologies (insulated water storage container; sani-scoop, potty, double-pit, pour-flush latrine; handwashing station, soapy-water storage bottle), and supplies (chlorine tablets, lipid-based nutrient supplements, laundry detergent sachets) integrated with parallel behavior-change promotion. Behavioral objectives were drinking treated, safely stored water, safe feces disposal, handwashing with soap at key times, and age-appropriate nutrition behaviors. We administered monthly surveys and spot-checks to households from randomly selected clusters for 6 months early in the trial. If any fidelity measures fell below set benchmarks, a rapid response mechanism was triggered.

**Results:**

In the first 3 months, functional water seals were detected in 33% (14/42) of latrines in the sanitation only arm; 35% (14/40) for the combined WSH arm; and 60% (34/57) for the combined WSH and Nutrition arm, all falling below the pre-set benchmark of 80%. Other fidelity indicators met the 65 to 80% uptake benchmarks. Rapid qualitative investigations determined that households concurrently used their own latrines with broken water seals in parallel with those provided by the trial. In consultation with the households, we closed pre-existing latrines without water seals, increased the CHWs’ visit frequency to encourage correct maintenance of latrines with water seals, and discouraged water-seal removal or breakage. At the sixth assessment, 86% (51/59) of households were in sanitation only; 92% (72/78) in the combined WSH; and 93% (71/76) in the combined WSH and Nutrition arms had latrines with functional water seals.

**Conclusions:**

An intensive implementation fidelity monitoring and rapid response system proved beneficial for this efficacy trial. To implement a routine program at scale requires further research into an adaptation of fidelity monitoring that supports program effectiveness.

**Trial registration:**

WASH Benefits Bangladesh: ClinicalTrials.gov, ID: NCT01590095. Registered on 30 April 2012.

## Background

Researchers typically report on the health impact and/or behavior change of large-scale, often complex public health interventions that include methods to persuade target groups to adopt an innovation such as a health-related technology or behavior [1]. Intervention impact may vary depending on programmatic efficiency, and political and social contexts. Contextual factors in both implementation and operation may moderate the impact of a planned intervention [2]. Very few community trials have reported on implementation fidelity, i.e., the degree to which an intervention is delivered as intended [3–5].

Implementation fidelity is an important fundamental tool for assessing the implementation process [6–8] which can help to explain the association between intervention and outcomes [4]. When limited or no impact is detected in an intervention trial, it is difficult to determine whether this occurred because the intervention was ineffective, or intervention uptake and implementation quality was poor [9]. Fidelity measurement can additionally enhance the credibility of intervention impact and transferability to other settings [10]. Conceptually, implementation fidelity has been defined using five elements: adherence to the planned intervention, exposure or dose, quality of delivery, participant responsiveness and program differentiation [4, 5]. Of these, quality of delivery can be measured against benchmarks set by the intervention implementers, to relate to adequate participant exposure [4, 11]. Determining whether fidelity indicators meet benchmarks early in the implementation period can guide corrective action through investigations of shortfalls in intervention delivery or uptake.

Among components of water, sanitation, and hygiene (WASH) interventions, there are some that can be initiated at one time point; e.g., water treatment or handwashing. However, sanitation interventions that include latrine construction require a series of steps to be completed; for example, negotiating location with the householders, digging the pit, producing the cement rings and slab, installing rings and slabs, and installing the above-ground (super) structure. Not all steps are necessarily completed as indicated; latrines may be half-constructed, or constructed and never used, or modified post construction. These conditions can limit the ability of latrines to separate feces from the environment, resulting in low implementation fidelity. WASH Benefits was a large-scale, cluster-randomized efficacy trial that aimed to assess the impact of improvements in water quality, sanitation, handwashing, and child nutrition and feeding – alone and in combination, on child health [12]. Therefore, it was necessary to achieve high levels of uptake of both enabling technologies (latrines, handwashing stations, etc.) and the related behaviors. To this end, we put in place an intensive system for monitoring intervention delivery, described in detail elsewhere [13]. Here, we describe development and use of fidelity measure indicators for the WASH Benefits Bangladesh study, as part of a three-paper series on WASH Benefits Intervention Delivery and Performance [13, 14]. We illustrate the benefits of early review of a rigorous, real-time fidelity assessment for this trial that required complex hardware delivery and installation, as well as parallel behavior-change promotion. We describe the use of benchmarks to signal early implementation problems, particularly for latrine installation that required qualitative investigation and course correction. Monitoring fidelity indicators throughout the intervention period is described in the third paper of this series [14].

## Methods

### WASH Benefits intervention design

The WASH Benefits Bangladesh randomized controlled trial as conducted across 720 clusters comprising six to eight geographically proximate households identified to have a pregnant mother at enrollment, totaling 4169 intervention households in four districts. Details of the study design are described elsewhere [12]. In summary, there were six intervention arms that included free provision of enabling technologies and supplies depicted in Table 1, integrated with parallel behavior-change promotion. The arms were (1) drinking-water treatment and safe storage, (2) sanitation, (3) handwashing, (4) child nutrition, (5) water, sanitation, handwashing (WSH), and (6) nutrition plus WSH. Locally recruited community health workers (CHWs), residents of the study villages, were trained to deliver and promote the interventions during regular home visits. Each CHW was responsible for one cluster of one intervention arm. Six intervention and two control geographically matched clusters were grouped into a trial block.

**Table 1:** Description of WASH Benefits trial interventions, fidelity measures and benchmarks, Bangladesh, 2012–2015

Interventions^a^	Provided to eligible households^b^	Fidelity measures^c^ (benchmark for uptake^c^)
Water quality	Free supply of chlorine tablets (Aquatabs® brand, sodium dichloroisocyanurate) with a 10-L insulated water storage container (Fig. 1a) Storage container, *N* = 2160 Tablets: 64,800/month	Presence of chlorine in stored water detected using a HACH color-wheel colorimeter (> 0.2 mg/L) (HACH LANGE GmbH, USA) (65%)
Sanitation	Double-pit improved latrine; sani- scoop, a locally developed tool for child and animal feces handling and disposal (Sultana et al.); potties (Faruqe) (Fig. 1b) Latrine installed: 4533 Child potty: 4384 Sani-scoop: 6303	Reported location and method of disposal for child’s last bowel motion (65%) Spot check for sani-scoop that easily accessible to mother (80%) Spot check for visible potty (easily accessible to mother) (80%) Spot check of designated household latrines; a functional water seal (80%)
Handwashing	A handwashing station comprised of a bucket with a fitted tap (40 L near the latrine and 16 L near the cooking area), a soapy-water bottle (Hulland et al. [18]) and free bi-weekly supply of detergent for soapy-water preparation (Fig. 1c) Station for latrine and kitchen: 4320	Spot check for handwashing location near the toilet and the cooking area, and for the presence of water and soap (65% with at least one with soap and water present)
Child nutrition	Promotion of exclusive breastfeeding up to 6 months of age, complementary feeding and supplementation using lipid-based nutrient supplements (LNS; Nutriset, France) for children aged 6–24 months (Fig. 1d). Total sachets distributed: 1,579,900	Reported messages on infant/child nutrition and/or lipid- based nutrient supplements (LNS) local name; Sonamoni (80%) Inspection of number of sachets of Sonamoni for children > 6 months of age, consistent with use of 2 sachets per day (70%)

^a^Combined arms: The interventions were delivered sequentially in the following order: sanitation, handwashing. Water treatment, with a minimum of 21 days between each start date. LNS was delivered when the child reaches 6 months of age; fidelity indicators and benchmarks as for individual components; ^b^eligible households were those directly enrolled by the study in the individual water, handwashing, and nutrition arms. For arms that received the sanitation intervention, interventions were provided to all households in the compound of the index household; potties were provided to all households in the compound of the index household who had children too young to use a latrine. ^c^based on unannounced visits by the Fidelity Assessment Team; does not include hardware replacements made during the course of the trial

Among households in the individual and combined sanitation arms, a sanitation team (comprising a research scientist, sanitation engineers from the implementing agency, village education resource center (VERC) and field attendants) installed study-provided latrines, independent of other intervention hardware delivery, with the first latrine installed in December, 2012 and the final in February, 2014. The latrine model, referred to as “double pit” (alternatively named dual pit, twin pit, two-pit, Fossa Alterna) is a traditional improved pit latrine that comprises two unconnected pits; one in use and one for which households transfer the above-ground (super) structure when the first pit is full [15]. This model uses a water seal (siphon), leading from the latrine to the pit that prevents odors from coming from the pit below into the toilet area (Fig. 2). The installation process involved multiple quality assurance steps. Latrine components were manufactured by pre-approved suppliers that met project-set standards of production; a subset of components was checked for compliance by breaking 5% of the concrete rings, slab and lid purchased for the project with a hammer. We checked the metal reinforcement of the same components by using metal detectors. If any evaluated component failed the quality check, the entire lot was rejected. The sanitation team used a checklist to verify complete latrine installation.

**Fig. 1:**
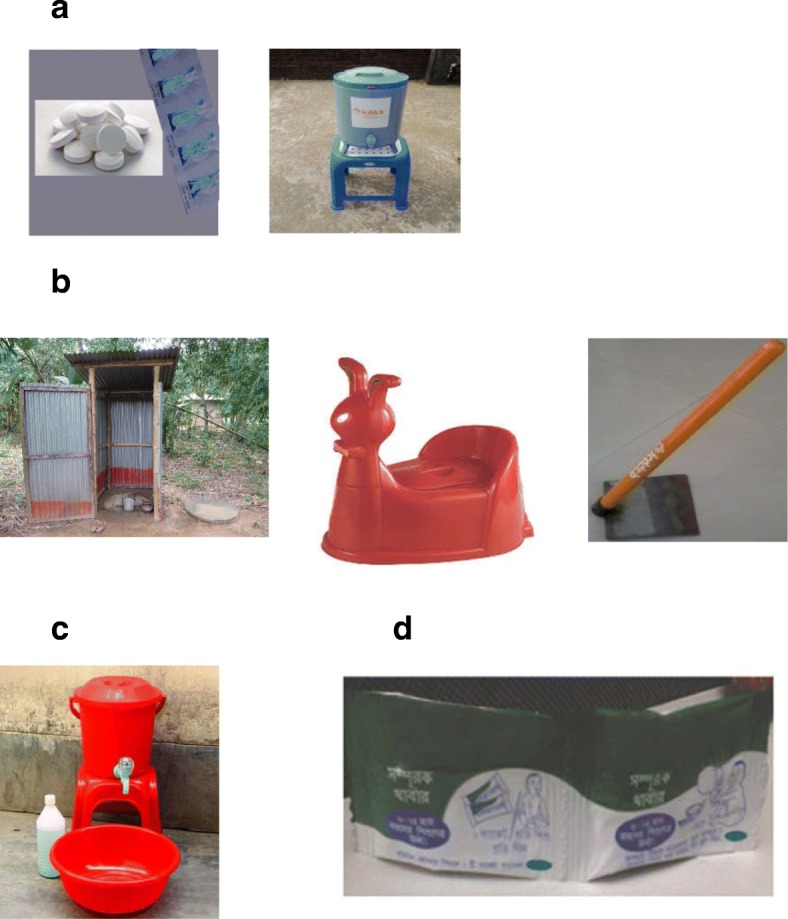
WASH Benefits interventions products. **a** chlorine tablets and water storage container. **b** double-pit, pour-flush latrine, child potty and sani-scoop. **c** handwashing station. **d** lipid-based nutrient supplement (LNS)

The enabling technologies for other intervention components included child potties [16], feces collecting sani-scoops [17], insulated drinking-water storage containers, handwashing stations, and soapy-water storage bottles [18]. Supplies provided by the project included laundry detergent sachets for soapy-water preparation, chlorine tablets (sodium dichloroisocyanurate, Aquatabs®, Medentech, Wexford, Ireland), and lipid-based nutrient supplements (LNS, Nutriset, Malauanay, France) (Table 1, Fig. 1). Behavioral objectives were drinking treated, safely stored water, safe feces disposal, handwashing with soap at key times, and age-appropriate nutrition behaviors (pregnancy to 24 months). The interventions were rolled out from September 2012 to the first 10 geographically proximate trial blocks, monitored and refined during a 2–3 month period, then rolled out to the remaining trial blocks in nine phases to accommodate the logistics and the large number of staff members needed to deploy quality interventions [13].

### Development of fidelity criteria and critical benchmarks

**Fig. 2:**
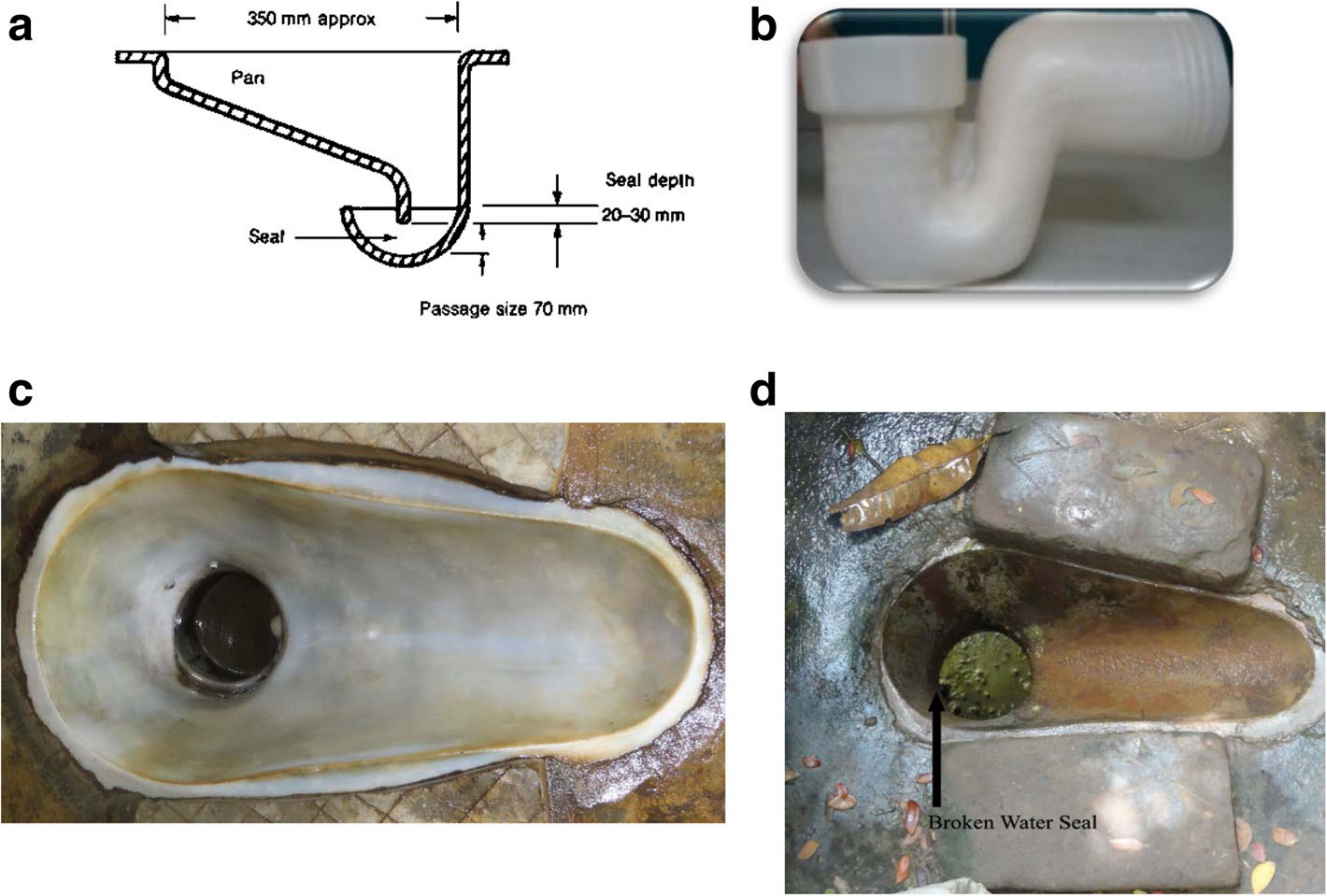
Latrine water seal: schematic diagram and example of a broken water seal; **a** diagrammatic cross section of the water seal. **b** photo of water seal model in WASH Benefits latrines. **c** with intact water seal (water is visible). **d** with broken water seal recognized because of the ability to see feces

Fidelity measurements were designed to capture the quality of intervention delivery, which we measured as the presence of fully functional hardware, exposure to CHW visits and indicators of technology and supply use. A team comprising the principal and co-investigators, international collaborators, local scientific staff and implementation specialists developed a priori fidelity indicators that could be easily measured, and critical benchmarks that could be feasibly attained (Table 1). These included observations of the presence, functionality, condition and signs of use of delivered hardware using a structured evaluation. For double-pit latrine functionality this included observation of an intact functional water seal (Fig. 2, Table 1). We conducted qualitative studies to develop specific definitions of ‘easily accessible to mother,’ defined as producing technologies/supplies within 10 s for child potties, sani-scoops, or the stock of LNS sachets, and checked for adequate delivery. These fidelity indicators and benchmarks were also used to monitor uptake throughout the trial, result of which are reported elsewhere [14].

### Data collection and analysis

We administered surveys and spot checks during unannounced fidelity assessment visits every month, from November, 2012, 2 months after intervention delivery commenced, and for the next 6 months. We surveyed more intensely in the first 4 months, all intervention households from four randomly selected blocks per month, of the 10 initial trial blocks where the interventions had already been delivered (*n* = 8 × 6 × 4 households). We surveyed four randomly selected households per intervention cluster from eight randomly selected blocks per month, of 20 trial blocks where the interventions had already been delivered (*n* = 4 × 6 × 8 households), in the remaining months. The sample size for assessing intervention fidelity was calculated based on feasibility and convenience, no statistical method was applied. We randomly selected the households each month to ensure representativeness. Measures were made more intensely in the early period because WASH Benefits was a large complex intervention with inevitable deviations from optimal implementation. We aimed for early identification of problem to guide corrective measures to avoid deviation in the subsequent intervention blocks.

We conducted both surveys and spot checks; the survey provided a snapshot of the attitudes and behaviors – including thoughts, meaningful opinions, and comments among the target population. Spot checks, conducted in real time, provided objective measures of hardware functionality and use indicating on the ground program operation, and any unexpected developments affecting the program and its objectives.

Field workers collected data on smartphones, using a survey and spot-check form on the presence (double-pit latrine with super structure, slab/pan, sani-scoop and potty for three or more children), functionality (double-pit latrine: superstructure is in good condition, slab/pan is in good condition, feces have not flowed out, functional water seal; potty and sani-scoop: not broken and easily accessible) condition and signs of use (double-pit latrine: wet floor and pan, water in water seal, feces in pan/water seal, pathway to latrine suggest regular use – is clear, without grass or any barrier, water container present; potty: visible sign of feces inside/on the pot/removable pot, sani-scoop: visible sign of feces on the sani-scoop, not broken)of provided technologies, and on reported corresponding behaviors (use of double-pit latrine, use of potty and sani-scoop, disposal practices for child and animal feces) (Table 1). Data collectors checked for completeness and consistency of forms after each visit. Data were stored in a secure server in a SQL database, and checked for validity and data consistency. We calculated frequencies of fidelity measures for each intervention component (Table 1) using STATA 13 (StataCorp LP, College Station, TX, USA).

The data were rapidly assessed and circulated as standardized reports within 30 days of data collection. If any of the monthly fidelity measures were below critical benchmarks, then a rapid response mechanism was triggered. Investigators including anthropologists trained in qualitative investigation and not involved in project implementation reviewed monitoring and process documentation previously filed from routine monthly field staff and CHW meetings in the low-performing areas. They also visited sites, conducted informal discussions with CHWs, their supervisors, field staff, and study participants to identify the underlying problem(s). The findings were summarized and discussed to develop appropriate actions in response. These included additional classroom training and on the job training for CHWs, their supervisors, and field staff, on how to problem-solve to achieve the pre-set benchmark.

## Results

### Intervention fidelity achievements

**Fig. 3:**
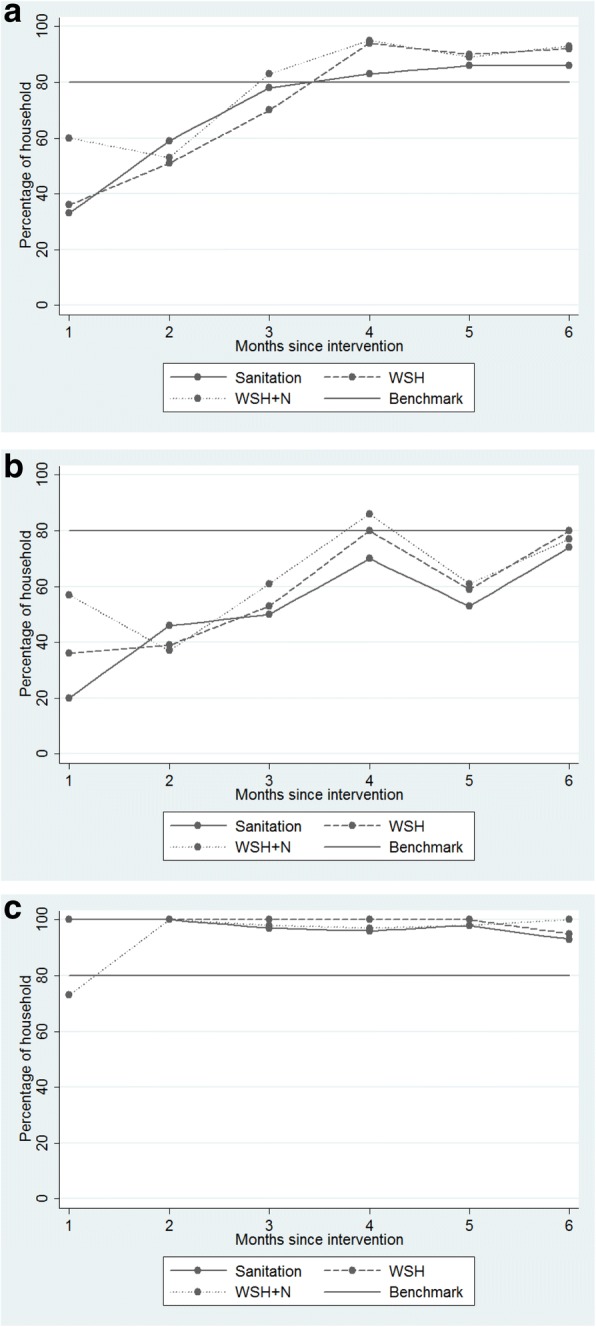
Sanitation fidelity measures (% households *n* = 176) per month, for first to sixth monthly assessments, and a priori benchmark, WASH Benefits, Bangladesh 2012–2015. **a** households in which latrines were found with an intact water seal (both existing and icddr,b). **b** households in which pre-existing latrines were found with an intact water seal. **c** households in which trial-provided latrines were found with an intact water seal

By the first month, the water treatment fidelity indicator, residual chlorine in stored water, met the benchmark (65%), as did the reported safe feces disposal indicator (critical benchmark 65%), sani-scoop easily accessible to the mother (80%) and reporting a CHW dissemination visit in the preceding month (90%; data not shown). At least one handwashing station with soap and water present (65%) was attained by the third month. Among households with children > 6 months of age, the benchmark for observable stocks of lipid-based nutrient supplements (LNS) consistent with use of two sachets per day (80%) was met when the child became eligible to consume it (fifth and sixth assessment), as was hearing messages on infant/child nutrition and LNS (70%; data not shown). Reported child feces disposal (last child defecation event disposed into the latrine or a designated pit) met the targeted benchmark (65%) at each fidelity assessment round. However, in three clusters, at the 1- and 3-month assessments, following the distribution of potties to all children under the age of 3 years living in the target household’s compound, this indicator was below the benchmark. Moreover, the sanitation benchmark of 80% of household latrines with a functional water seal was not met by the fourth assessment (Fig. 3a).

### Investigation of shortfall in sanitation implementation benchmarks: Latrine maintenance and use

The reasons for which < 80% of household latrines were found with a functional water seal were explored from the third assessment month by staff responsible for latrine installation, the qualitative team, CHWs and their supervisors. The qualitative investigation found that several households intentionally removed or broke the water seals in the newly installed, trial provided latrines (Figs. 2 and 3b). This is because they perceived that the water seals required extra water to flush the fecal contents around the S-shaped bend into the pit below and might fill the pits rapidly, blocking easy flow of feces into the pit, or causing water to splash during defecation. The recipients did not pour an adequate volume of water into the toilet during post-defecation cleansing, and thus failed to dislodge the fecal material completely from latrine pans, leading to low usage rates and poor maintenance (breaking/removing water seals). The new latrines were, therefore, less convenient than previously owned unimproved pit latrines. Furthermore, household members reported that they continued to use their existing latrines out of habit, kept them for visitors, outsiders and neighboring non-recipient households, many lacking a functional water seal (Fig. 3c) despite the availability of trial-provided improved latrines.

The qualitative team worked with CHWs and the latrine installation team to address these issues. In home visits and courtyard meetings, they demonstrated how the siphon/water seal functioned, and stressed the need to pour additional water with force to flush the feces through the water seal. They also discussed the advantage of having a functional water seal, citing reduced bad odors, and fewer flies and mosquitoes coming from the pit while a person was using the toilet. To discourage continued use of previously existing “unhygienic” (lacking functioning water seal) latrines, they were structurally closed with the household’s consent to prevent ongoing environmental contamination.

### Investigation of shortfall in sanitation implementation benchmarks: Unsafe child feces disposal

Prior to potty distribution, > 90% children aged < 3 years were habitually defecating in the courtyard (Mahfuza Islam, manuscript in preparation). Qualitative investigation revealed that although potties were provided and promoted according to protocol guidelines, additional support was required to encourage and teach rural mothers how to train their children to *consistently* use the potty. In addition, CHWs encouraged the mothers to store potties in a place that was accessible to the trained toddler when they need to use it; they encouraged household members other than the mother to participate in the potty training and habit formation. In addition, we provided refresher training to the CHWs to address safe feces disposal into latrines or covered pits, instead of the prevalent practices of disposing feces into the bushes just out of sight.

## Discussion

We have summarized the implementation fidelity system put in place for the WASH Benefits Bangladesh efficacy trial, and provided an example of how systematic fidelity measures can detect compliance and deviations and trigger a rapid course correction. Such careful monitoring is especially essential in complex community-based efficacy studies where high uptake of technologies and behaviors is necessary to achieve the study aims [13]. Deviations in fidelity for large-scale interventions can also be detected through internal monitoring and evaluation, but this often suffers from slow report turnaround and insufficient review for critical feedback. An objective fidelity review with rapid assessment of initial rollout can be done in parallel to implementation process documentation and monitoring, to provide critical input and focus resources for increased efficiency.

Complex interventions tend to have lower fidelity because they are comparatively more difficult to implement than simpler interventions [1, 19]. This is consistent with our findings for the sanitation intervention in the WASH Benefits Bangladesh study that included labor-intensive, multi-stepped latrine installation, close supervision, and quality assurance. The sanitation team installed 4533 latrines, for enrolled households and their neighbors in 21 sub-districts across four districts over 15 months. In our case, fidelity measurements demonstrated that the intervention was implemented as intended by the sixth assessment month; however, this required qualitative investigation and adjustments to the respective behavioral-change package.

Providing enabling products can encourage target behaviors; product quality influences uptake or response among intervention recipients [20]. In our case, households found the project-provided latrine of good quality but less convenient when supplied with a water seal, which some subsequently broke or removed. Breaking or removing water seals has been observed in Bangladesh [21] and India [22] and is believed to be common; however, there are few published data on the frequency of this practice. Determining how commonly this occurs could benefit behavior-change efforts in future latrine interventions in South Asia and among low-income countries where this practice may occur. Latrines with broken or missing water seals have a reduced ability to keep excreta separate from the environment and as such are categorized as unimproved [23].

We did not develop an implementation fidelity score or index to communicate the results in the monthly reports. A numerical index or score calculated for each assessment round might be useful in demonstrating the level of fidelity across combined intervention arms compared to individual intervention arms in a multi-factorial design as in WASH Benefits. However, the specific objective of early fidelity measures was to trigger investigation to address low uptake. A fidelity or implementation index may be relevant for explaining intervention uptake over the 24-month intervention period, to assist interpreting health impact.

In summary, fidelity indicators were efficient, quick to collect, easily observable and indicated the presence, functionality of hardware, condition and sign of use, and corresponding behaviors, which focused on technology accessibility, behavior-change communication crucial for sustained intervention uptake. Only the indicators for child feces disposal and CHW-delivered nutrition messages were reported and not directly observed and were, therefore, potentially overestimated. Fidelity assessment was conducted by only three research assistants who were not directly involved in intervention delivery thus data were generated at relatively low cost. Fidelity indicators described here can be used in other similar intervention trials.

## Conclusions

An intensive implementation fidelity monitoring and response system proved beneficial for the WASH Benefits efficacy trial. Timely exploration of benchmark shortfalls enabled corrective measures to ensure that implementation adhered to the intended protocol. The indicators used were derived from easily observable, easy-to-collect data that can be used in other similar WASH and nutrition trials. Flexible adaptations to behavioral interventions at the community level can be directed by time-sensitive fidelity assessments and need to be documented to inform future large-scale studies in similar settings.

Although fidelity assessments are often conducted during the pilot or developmental stages of a study, the integrity of research studies of all designs and stages would benefit from a structured fidelity assessment as a key component [19, 24]. To implement a routine program at scale requires further research into an adaptation of fidelity monitoring that supports program effectiveness.

## Abbreviations

BCC: Behavior-change communication
CHW: Community health worker
VERC: Village education resource center
WSH: Water, sanitation, and hygiene
